# The prognostic value of prognostic nutritional index in postoperative onset of PAH in children with isolated VSD: a prospective cohort study based on propensity score matching analysis

**DOI:** 10.3389/fped.2024.1292786

**Published:** 2024-04-18

**Authors:** Zeying Zhang, Jing Su, Chenyang Li, Shirui Cao, Chao Sun, Qiuzhen Lin, Haiyan Luo, Zhenghui Xiao, Yunbin Xiao, Qiming Liu

**Affiliations:** ^1^ Department of Cardiology, The Second Xiangya Hospital of Central South University, Changsha, China; ^2^ Department of Cardiology, Hunan Children's Hospital, Changsha, China; ^3^ Class 2115, Yali High School, Changsha, China; ^4^ Department of General Ward for Critical Illness, Hunan Children’s Hospital, Changsha, China; ^5^ Department of Intensive Care Unit, Hunan Children’s Hospital, Changsha, China

**Keywords:** pulmonary arterial hypertension, prognostic nutrition index, propensity score matching, prognosis, ventricular septal defect, pediatric

## Abstract

**Background:**

The mechanism of pulmonary arterial hypertension (PAH) after surgery/intervention for isolated venticlular septal defect (VSD) in children is unknown. Reliable prognostic indicators for predicting postoperative PAH are urgently needed. Prognostic nutration index (PNI) is widely used to predict postoperative complications and survival in adults, but it is unclear whether it can be used as an indicator of prognosis in children.

**Methods:**

A total of 251 children underwent VSD repair surgery or interventional closure in Hunan Children's Hospital from 2020 to 2023 were collected. A 1:1 propensity score matching (PSM) analysis was performed using the nearest neighbor method with a caliper size of 0.2 Logistics regression analysis is used to examine factors associated with the development of PAH.

**Results:**

The cut-off value for PNI was determined as 58.0. After 1:1 PSM analysis, 49 patients in the low PNI group were matched with high PNI group. Children in the low PNI group had higher risk of postoperative PAH (*P* = 0.002) than those in the high PNI group. Multivariate logistics regression analysis showed that PNI (RR: 0.903, 95% CI: 0.816–0.999, *P* = 0.049) and tricuspid regurgitation velocity (RR: 4.743, 95% CI: 1.131–19.897, *P* = 0.033) were independent prognostic factors for the development of PAH.

**Conclusion:**

PNI can be used as a prognostic indicator for PAH development after surgery/intervention in children with isolated VSD.

## 1 Introduction

Congenital malformations are an important cause of death in children whose important component is congenital heart disease (CHD) (1). Congenital heart disease is considered the most common and serious abnormality at birth, with a prevalence of 6–13 cases per 1,000 births (2, 3). Venticlular septal defect (VSD) is a congenital heart disease that causes communication between the left and right ventricles due to embryonic ventricular septal dysplasia, resulting in horizontal ventricular shunting, accounting for about 40 percent of congenital heart disease and is the most common congenital heart disease (4, 5).

Surgery is an effective treatment for children with VSD which includes repair surgery and interventional closure. The development of pulmonary arterial hypertension (PAH) is an important factor affecting the prognosis of children after VSD surgery. Pulmonary hypertension crises lead to significantly increased perioperative mortality in children, and on the other hand, life expectancy in VSD patients with postoperative PAH is even lower than in patients with unoperated complex VSD and Eisenmenger syndrome (6). Therefore, early screening of patients who may have PAH after surgery/intervention is of great significance to improve the success rate of treatment. Recognized risk factors influencing the development of PAH after VSD repair surgery or interventional closure include age, defect size, and pulmonary vascular resistance (7). However, these factors did not take into account the nutritional status of the child.

Malnutrition is a common problem in children born with dysplasia or prematurity. Malnutrition can affect the patient's response to treatment, resulting in a poorer quality of life. Assessment of systemic nutritional status is achieved by introducing the prognostic nutration index (PNI), a continuous variable based on serum albumin concentration and total peripheral blood lymphocyte count (8). PNI was originally designed to assess perioperative immunotrophic status and surgical risk in patients undergoing gastrointestinal surgery (9). Several studies have demonstrated the prognostic value of PNI in a variety of malignancies and in adult patients with pulmonary hypertension (10–12). However, it is unclear whether it is an indicator of the prognostic development of PAH in children after VSD repair surgery or interventional closure.

Confounding factors always impair the accuracy and objectivity of cohort studies. Propensity score matching analysis (PSM) is commonly used to overcome selection bias, adjust for confounders, improve comparability between groups by increasing the level of evidence in cohort studies, and simulate randomization of observed covariates (13–15). This prospective cohort study first performed PSM analysis to assess the prognostic value of PNI for postoperative PAH development in children with acute VSD repair or interventional closure.

## 2 Materials and methods

### 2.1 Ethics statement and cohort selection

This is a prospective cohort study of children who attended the Department of Cardiology of Hunan Children's Hospital from January 2020 to January 2023. The study was approved by the Ethics Committee of Hunan Children's Hospital. Written informed consent signatures were obtained from the parents of children included in the studies, and all data were de-identified for analysis. Children undergoing VSD repair surgery or interventional closure who met the following inclusion criteria were included in the study: (1) transthoracic echocardiography for isolated ventricular septal defect without other cardiac malformations; (2) did not undergo any cardiac surgery or interventional procedures prior to admission; (3) were aged 3 months–18 years; (4) had complete clinical data and detection indicators. Exclusion criteria: (1) concurrent pulmonary hypertension caused by left heart disease, lung disease, hypoxia, chronic thromboembolic pulmonary hypertension or other diseases within 1 week before surgery/intervention; (2) incomplete case data; (3) unwilling to participate in the investigation. All procedures followed were in accordance with the ethical standards of the responsible committee on human experimentation (institutional and national) and with the Helsinki Declaration of 1975, as revised in 2000.

### 2.2 Data collection

Pediatric clinical data were collected from electronic medical records. During admission, the following variables were considered: age, sex, body mass index (BMI), VSD defect size, preoperative New York Heart Association (NYHA) functional class, postoperative development of PAH, echocardiographic indicators including tricuspid regurgitation velocity (TRV), right ventricular end-diastolic diameter (RVEDd), left ventricular end-diastolic diameter (LVEDd) and right atrial diameter (RAd), electrocardiography (PR interval, QRS wave) at 1 week before surgery/intervention, and serum albumin and complete blood lymphocyte count within 1 week before surgery/intervention. VSD defect size is divided into 3 grades. Small defects are those with a defect diameter of less than 5 mm; defect of medium size refers to the defect diameter between 5 and 9 mm; Large defects are those that are larger than 9 mm in diameter. The preoperative NYHA functional class has four grades: Grade I refers to unrestricted physical activity. Preschoolers are able to participate in physical education classes and to move around the same as children of the same age in this grade. Grade II refers to mild limitation of physical activity without any discomfort at rest, but general activity can cause fatigue, palpitations, or dyspnea. School-age children are able to participate in physical education but are less active than children their age and may have secondary growth disorders in this grade. Grade III refers to obvious limitation of physical activity, less than usual general activities can appear symptoms. School-age children cannot participate in physical activities and there are secondary growth disorders in this grade; Grade IV refers to the inability to engage in any physical activity, heart failure symptoms at rest, and worsening after activity, and secondary growth disorders. TRV is divided into 3 grades. The low tricuspid regurgitation velocity tricuspid is <2.8 m/s. The modorate tricuspid regurgitation velocity is 2.8–3.4 m/s. The remaining is moderate grade.

### 2.3 Follow up and endpoints

We followed all children until 31 August 2023 or when PAH developed. Postoperative follow-up examination of hemodynamics lasted for more than half a year. The diagnosis of PAH meets the criteria of the 2021 Guidelines for the Diagnosis and Treatment of Pulmonary Hypertension Associated with Congenital Heart Disease in Children (16): mean pulmonaryartery pressure (mPAP) >20 mmHg measured by standard right heart catheterization, pulmonary artery wedge pressure (PAWP) ≤15 mmHg, pulmonary vascular resistance index (PVRI)≥ 3 WU·m^2^. If right heart catheterization was not performed, TRV >3.4 m/s is measured by echocardiography.

### 2.4 Assessment and calculation of PNI

PNI is a scoring system that reflects a patient's nutritional and immune status. It is calculated based on serum albumin and lymphocyte. PNI = albumin (g/L) + 5 × lymphocyte count (10^9^/L).

### 2.5 Statistical analyses

Data for continuous variables that conform to a normal distribution are expressed as mean (standard deviation, SD) and otherwise by median (interquartile range, IQR). Data for categorical variables are expressed as numbers (percentage, %). The comparison of continuous variables that do not conform to the normal distribution is analyzed by rank-sum test (Mann-Whitney test), otherwise the t-test is used. The *χ*^2^ test was used to analyze the differences in clinical factors. The nearest neighbor method was used for 1:1 PSM to reduce selection bias and confounding factors caused by different covariate distributions between low and high PNI groups. Matching factors included clinical features of baseline unequivalence between the two groups, including age, gender, BMI, NYHA functional class, VSD defect size, TRV, RAd, RVEDd, NT-pro BNP and PR interval. The caliper size is 0. 2 to determine the effect of PNI on the development of PAH in children with isolated VSD after surgery/intervention. After the 1:1 propensity score matching, continuous variables were compared using the Mann-Whitney *U*-test and categorical variables were compared using the *χ*^2^ test. The logistics model was used to analyze the univariate correlation between prognostic factors and postoperative PAH development. In multivariate analysis, all variables with *P* < 0.1 in univariate analysis are included in the model. All statistical tests were two-sided and statistically significant set to *P* < 0.05. All statistical analyses were performed using SPSS 26.0 software (IBM).

## 3 Results

### 3.1 Characteristics of included patients before and after PSM

143 children were excluded from this study due to concurrent pulmonary hypertension within 1 week before surgery/intervention. The characteristics of included 251 children with isolated VSD who underwent repair surgery or interventional closure are detailed in Table 1. The cut-off value is obtained as 58.0 according to the Receiver Operating Characteristic (ROC) curve, at which point the Jordon index reaches its maximum (Supplementary Figure S1). A PNI greater than 58.0 was in the high PNI group, and vice versa in the low PNI group. A total of 56 children were in the low PNI group and 195 in the high PNI group. The median age in the low PNI group was 2.3 (IQR: 3.1) years, of whom 28 (50.0%) were male. The median age of children in the high PNI group was 1.9 (IQR: 2.6) years, of whom 95 (48.7%) were male. Children in the high PNI group had a higher BMI (*P* = 0.025). A higher proportion of children in the low PNI group had high-grade NYHA functional class (*P* = 0.037), VSD defect size (*P* = 0.005), and TRV (*P* = 0.003). NT-pro BNP (*P* = 0.012) was larger in the low PNI group than those in the high PNI group. A greater proportion of children in the low PNI group developed PAH underwent VSD after surgery/intervention (15.0% vs. 7.0%, *P* < 0.001).

**Table 1:** Associations between PNI and clinicopathological factors before and after PSM.

Variables	Before propensity matching		*P*	After propensity matching		*P*
	PNI ≤58	PNI >58		PNI ≤58	PNI >58	
	(*n* = 56)	(*n* = 195)		(*n *= 49)	(*n *= 49)	
Age (y)	2.3 (3.1)	1.9 (2.6)	0.431	1.9 (2.3)	1.9 (2.5)	0.964
Gender (male)	28 (50.0)	95 (48.7)	0.866	24 (49.0)	26 (53.1)	0.686
BMI	13.9 (2.0)	14.7 (2.3)	0.025*	13.9 (2.0)	13.9 (2.2)	0.114
NYHA functional class			0.037*			0.997
I	16 (28.6)	112 (42.6)		14 (28.6)	19 (30.6)	
II	26 (46.4)	144 (46.7)		24 (49.0)	40 (46.9)	
III	10 (17.9)	22 (7.7)		9 (18.4)	6 (18.4)	
IV	14 (7.1)	7 (3.1)		2 (4.1)	5 (4.1)	
Defect size			0.005*			0.226
Small	3 (5.4)	27 (13.8)		3 (6.1)	1 (2.0)	
Medium	12 (21.4)	73 (37.4)		12 (24.5)	19 (38.8)	
Large	41 (73.2)	95 (48.7)		34 (69.4)	29 (59.2)	
TRV			0.003*			0.507
Low	34 (60.7)	156 (80.0)		33 (67.3)	36 (73.5)	
Modorate	22 (39.3)	39 (20.0)		16 (32.7)	13 (26.5)	
RAd	20.8 (7.0)	20.4 (7.0)	0.671	20.8 (5.3)	19.2 (7.0)	0.863
RVEDd	18.3 (4.0)	18.1 (4.0)	0.746	18.2 (4.2)	17.3 (5.0)	0.646
RVEDd/LVEDd	0.62 (0.15)	0.60 (0.14)	0.355	0.62 (0.15)	0.58 (0.15)	0.830
NT-pro BNP	4,193.2 (4,405.6)	1,499.7 (1,979.0)	0.012*	2,335.6 (2,731.6)	3,027.5 (4,644.8)	0.754
PR interval	112.5 (21.7)	110.1 (24.0)	0.438	110.9 (22.0)	110.9 (32.0)	0.762
QRS wave	84.5 (22.0)	83.82 (14.0)	0.912	82.2 (20.0)	95.2 (26.0)	0.244
PAH	15 (26.8)	7 (3.6)	<0.001*	11 (22.4)	1 (2.0)	0.002*

BMI, body weight index; NYHA, New York Heart Association; TRV, tricuspid regurgitation velocity; Rad, right atrial diameter; RVEDd, right ventricular end-diastolic diameter; LVEDd, left ventricular end-diastolic diameter; PAH, pulmonary hypertension.

* *p* < 0.05.

After PSM, a total of 49 children in the low PNI group were matched with a high PNI group. After PSM, there was a good balance between the two groups, as there was no longer a significant difference between each confounding factor (*P* > 0.05, Table 1). Patient characteristics after PSM are detailed in Table 1. After PSM, a higher proportion of children in the low PNI group developed PAH after VSD surgery/intervention compared with high PNI group (22.4% vs. 2.0%, *P* = 0.002).

### 3.2 Univariable and multivariable analysis of included patients

After PSM, univariate analysis showed that high PNI was a protective factor for postoperative PAH in children after VSD surgery/intervention (RR = 0.893, *P* = 0.022, Table 2). Children with moderate (*P* = 0.027) TRV are more likely to develop postoperative PAH than low TRV. Right atrial diameter (*P* = 0.037) was risk factors for postoperative PAH in children after VSD surgery/intervention. Multivariate analysis showed that high PNI remained an independent prognostic protective factor for postoperative PAH in children after VSD surgery/intervention (RR = 0.903, *P* = 0.049). The other factor include VSD defect size (*P* = 0.066), TRV (*P* = 0.033) and RVEDd (*P* = 0.067).

**Table 2:** Univariable and multivariable analysis after 1:1 ratio PSM.

Variables	Univariable analysis			Variables	Multivariable analysis		
	RR	95% CI	*P*		RR	95% CI	*P*
PNI	0.893	0.810–0.984	0.022*	PNI	0.903	0.816–0.999	0.049*
Age	1.070	0.886–1.291	0.484	Age			
Gender (male)	0.435	0.122–1.533	0.200	Gender (male)			
BMI	0.911	0.609–1.363	0.651	BMI			
NYHA functional class			0.303	NYHA functional class			
I	reference			I			
II	4.098	0.467–35.918	0.203	II			
III	8.000	0.816–78.471	0.074	III			
IV	9.333	0.457–190.626	0.1447	IV			
Defect size (large)	0.139	0.017–1.127	0.065	Defect size (large)	7.934	0.875–71.939	0.066
TRV (modorate)	4.073	1.172–14.154	0.027*	TRV (modorate)	4.743	1.131–19.897	0.033*
RAd	1.138	1.008–1.286	0.037*	RAd			
RVEDd	1.162	0.995–1.356	0.058	RVEDd	1.17	0.989–1.400	0.067
RVEDd/LVEDd	1.692	0.028–103.693	0.802	RVEDd/LVEDd			
NT-pro BNP	1.000	1.000–1.000	0.655	NT-pro BNP			
PR interval	1.028	0.998–1.058	0.064	PR interval			
QRS wave	0.999	0.987–1.011	0.887	QRS			

BMI, body weight index; NYHA, New York Heart Association; TRV, tricuspid regurgitation velocity; RAd, right atrial diameter; RVEDd, right ventricular end-diastolic diameter; LVEDd, left ventricular end-diastolic diameter.

* *P *< 0.05.

## 4 Discussion

The development of PAH is an important factor affecting the prognosis of children after CHD surgery/intervention (17). As VSD is the most common congenital heart disease, early identification of VSD patients who may develop pulmonary hypertension after surgery/intervention is important to improve the success rate of treatment (18). In this prospective single-center cohort study, we analyzed the value of PNI in predicting postoperative PAH in children with VSD. For making the results more convincing, we used PSM analysis to reduce confounding effects, balance the differences of clinicopathological confounding factors for the groups. This method allows us to use non-randomized grouping data to estimate the relationship between PNI and postoperative onset of PAH. These clinicopathological confounding factors including age, gender, BMI, NYHA functional class, VSD defect size, TRV, RAd, RVEDd, NT-pro BNP and PR interval were well adjusted, and no differences were demonstrated between the low and high PNI groups after PSM. After PSM, factors including PNI and TRV remain independent association with the development of postoperative PAH in multivariate analysis model. To our knowledge, this is the first study to assess the effect of PNI on the prognostic effect of PAH after surgery/intervention for congenital heart disease by scoring matching analysis, and we demonstrated that PNI is an independent prognostic factor.

Nutritional status can affect the prognosis of PAH. Malnutrition is a complex problem with many causes. The causes of malnutrition in patients with PAH are multifactorial and include loss of appetite, malabsorption due to right heart failure, side effects of specific medications, increased metabolic rate, and dyspnea. Malnutrition is thought to be more likely to be the result of PAH. Studies have shown that children with young, low-weight congenital heart disease associated with pulmonary arterial heart disease have a higher incidence of PAH after surgery (19). It is thought that the possible cause is the large congenital heart disease defect in children with young age and low weight. Large defects cause a large number of left-to-right shunts that affect the blood circulation of organs and tissues, as well as the growth and development of the child, resulting in low body weight. In addition, large shunts due to large defects can lead to early pulmonary vascular remodeling and dynamic PAH. At the same time, large shunts may also hinder the transition of pulmonary circulation to adult form in some children after birth, and pulmonary artery pressure is persistently high. Thus, low body weight and malnutrition may be due to major defects and subsequent pulmonary hemodynamic disturbances and pulmonary vascular remodeling. However, this study found that nutritional status can influence the occurrence of postoperative PH in children with isolated VSD, not just the consequences of PAH. There may be a vicious circle between malnutrition and PAH. Therefore, nutritional status assessment is an important part of predicting prognosis. Assessment of nutritional status is multidimensional. Although nutritional indicators such as hypoalbuminemia are associated with the risk of mortality in patients with PAH (9), their single evaluation criteria limit the scope of application. Nutritional status can be assessed using the PNI, which combines an assessment of metabolic status and an assessment of inflammatory status. Inflammation and immune imbalance are thought to be involved in the development of pulmonary vascular remodeling in PAH, which is an important pathological feature of PAH (20). PNI is a viable tool to assess the relationship between immunotrophic status and prognosis and has been widely used in acute heart failure, esophageal cancer, and lymphoma (21–24). In fact, the prognostic value of PNI index for survival in adult patients with PAH has also been reported (12). Low PNI was associated with an increased risk of death in PH patients. However, the prognosis of whether PNI indices can be used for PAH in children is unclear. In this study, we used the PNI to assess the nutritional status of children with VSD after VSD surgery/intervention and confirmed that PNI is an independent prognostic factor predicting the development of PAH. The optimal cut-off point for PNI was determined to be 58.0. This value is larger compared to the results of other studies in adults (8, 25). This may be due to the shorter period of disease attrition in children than in adults.

TRV can be used as both a diagnostic and prognostic indicator of PAH.The use of TRV as a key variable in echocardiography for the diagnosis of PAH has been recommended (26). Studies have shown that there are different criteria for the diagnosis of pulmonary hypertension based on TRV in different inclusion criteria and study participants (27, 28). The 2022 ESC guidelines for pulmonary hypertension state that the likelihood of pulmonary hypertension is low when TRV ≤2.8 m/s, and that PAH should be highly suspected at 2.8 m/s <TRV <3.4 m/s (29). If TRV >3.4 m/s, PAH should be taten into consideration clinically (29). After balancing the confounding factors, this study found that TRV is associated with the development of PAH, and high TRV is more dangerous than low TRV. TRV marks a high afterload of the right heart, and its value also depends on the adaptive capacity of the right heart, which means TRV is not necessarily large when pulmonary artery blood pressure is high. RVEDd is also an important prognostic factor for the development of PAH. In the development of PAH, the right heart changes from compensatory hypertrophy to decompensatory hypertrophy and even failure in the process of adapting to increased afterload, accompanied by right heart fibrosis, metabolic disorders, apoptosis of cardiomyocytes and inflammatory damage (30, 31). The increase in RVEDd marks the right heart reconstruction process after increased afterload.

There are some advantages to this study. Firstly, this study proposes for the first time the application of PNI in predicting the development of PAH in children with CHD after surgery/intervention. Children with low PNI can be alerted early to the development of a PAH crisis and the development of PAH. Second, the laboratory indicators involved in PNI are simple, practical, and effective biomarkers in routine examinations in hospitalized children, because these indicators can be assessed by routine blood and liver function tests without causing greater financial pressure.

However, there are some limitations in this study that cannot be ignored. First, this was a cohort study design conducted at a single center with a limited number of patients. A multicentre study and more patients should be included. Second, selection bias could not be ruled out, even if consecutive patients were included and eligibility criteria were implemented to reduce bias. Third, PNI is a non-specific tumor marker that can be confused with other non-cancer and cancerous diseases. Further validation of a large prospective study is needed to further evaluate the prognostic and predictive value of future PNI for the development of PAH after VSD surgery/intervention.

## 5 Conclusion

In conclusion, by using propensity score matching analysis, we confirmed that adjusted PNI is a valid prognostic factor for the postoperative development of PAH in children with VSD. However, due to inherent flaws in the retrospective design, more prospective studies are needed to confirm this result in the future.

## Data Availability

The raw data supporting the conclusions of this article will be made available by the authors, without undue reservation.

## Supplementary material

Supplementary Figure S1 (**A**) PNI score is higher in patients with no PAH after surgery (*n* = 229) than in patients with PAH (*n* = 22; 65.65 vs 58.44, *P* = 0.003); (**B**) Receiver Operating Characteristic (ROC) curve shown that area under the curve (AUC) is 0.695 and *P* value of 0.003; (**C**) The Jordon index achieves its maximum when the PNI index is equal to 58.0.
