# Supplementary figures and images for: The prognostic value of prognostic nutritional index in postoperative onset of PAH in children with isolated VSD: a prospective cohort study based on propensity score matching analysis
Source: Front Pediatr. 2024 Apr 18;12:1292786. doi: 10.3389/fped.2024.1292786 (PMC11064175; doi:10.3389/fped.2024.1292786)

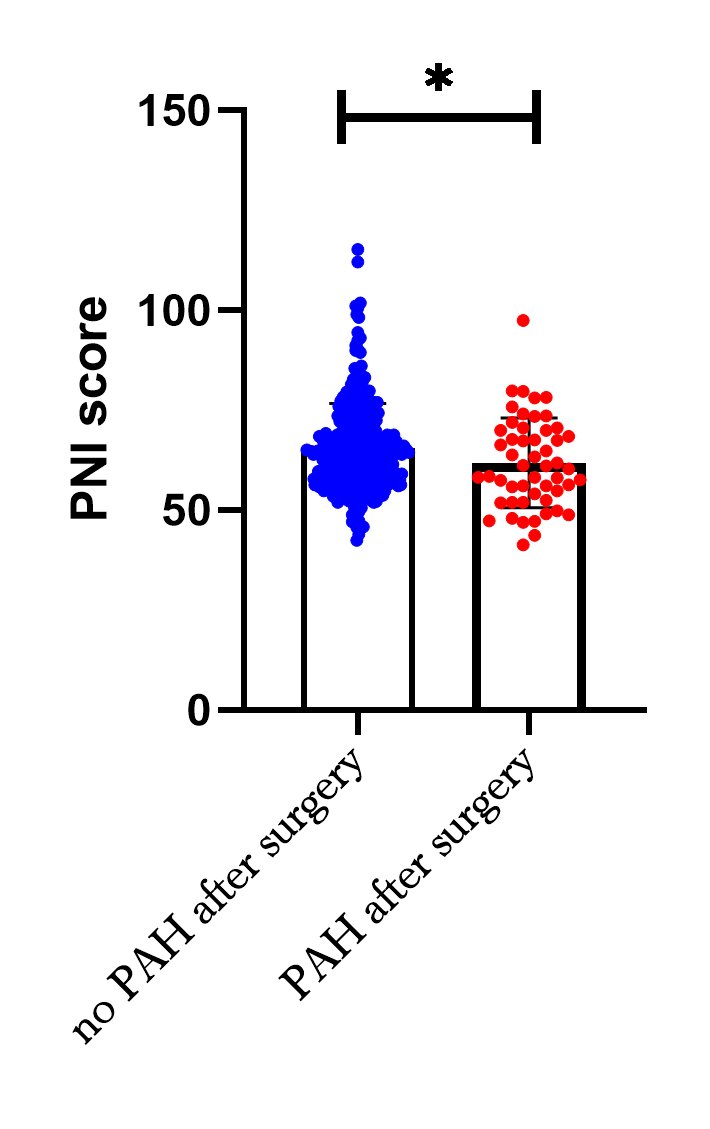

Supplement: Supplementary Figure S1 — (A) PNI score is higher in patients with no PAH after surgery (n = 229) than in patients with PAH (n = 22; 65.65 vs 58.44, P = 0.003); (B) Receiver Operating Characteristic (ROC) curve shown that area under the curve (AUC) is 0.695 and P value of 0.003; (C) The Jordon index achieves its maximum when the PNI index is equal to 58.0. [file Image1.tif]
